# A survey of the management of urinary tract infection in children in primary care and comparison with the NICE guidelines

**DOI:** 10.1186/1471-2296-11-6

**Published:** 2010-01-26

**Authors:** Kieran M Kennedy, Liam G Glynn, Brendan Dineen

**Affiliations:** 1 Department of General Practice, National University of Ireland, Galway, Ireland; 2 Clinical Science Institute, Galway University Hospital, Galway, Ireland

## Abstract

**Background:**

The aim of this study was to establish current practices amongst general practitioners in the West of Ireland with regard to the investigation, diagnosis and management of urinary tract infection (UTI) in children and to evaluate these practices against recently published guidelines from the National Institute for Health and Clinical Excellence (NICE).

**Methods:**

A postal survey was performed using a questionnaire that included short clinical scenarios. All general practices in a single health region were sent a questionnaire, cover letter and SAE. Systematic postal and telephone contact was made with non-responders. The data was analysed using SPSS version 15.

**Results:**

Sixty-nine general practitioners were included in the study and 50 (72%) responded to the questionnaire. All respondents agreed that it is important to consider diagnosis of UTI in all children with unexplained fever. Doctors accurately identified relevant risk factors for UTI in the majority (87%) of cases. In collecting urine samples from a one year old child, 80% of respondents recommended the use of a urine collection bag and the remaining 20% recommended collection of a clean catch sample. Respondents differed greatly in their practice with regard to detailed investigation and specialist referral after a first episode of UTI. Co-amoxiclav was the most frequently used antibiotic for the treatment of cystitis, with most doctors prescribing a five day course.

**Conclusions:**

In general, this study reveals a high level of clinical knowledge amongst doctors treating children with UTI in primary care in the catchment area of County Mayo. However, it also demonstrates wide variation in practice with regard to detailed investigation and specialist referral. The common practice of prescribing long courses of antibiotics when treating lower urinary tract infection is at variance with NICE's recommendation of a three day course of antibiotics for cystitis in children over three months of age when there are no atypical features.

## Background

Urinary tract infection (UTI) is a common condition in children. Approximately 1 in 10 girls and 1 in 30 boys will have a UTI by the age of 16 years [1]. Renal scarring as a result of UTI may lead to hypertension, decreased renal function, proteinuria and end-stage renal disease. This is especially true if the condition is not diagnosed, investigated and managed appropriately. However, UTI in children is a most challenging condition to treat in primary care because symptoms can be minimal in the early stages and urine samples are often difficult to obtain. Previously, the paucity of clear evidence-based clinical practice guidelines may also have contributed to diverse treatment approaches. The National Institute for Health and Clinical Excellence (NICE) has recently published new guidelines on the diagnosis, treatment and long-term management of UTI in children less than 16 years of age [2].

The aim of this study is to establish current practice amongst general practitioners working in a single health region in the West of Ireland with regard to the investigation, diagnosis and management of urinary tract infections in children and to compare this to NICE guideline recommendations. The authors are unaware of any similar published research in Ireland or in the United Kingdom since the publication of this guideline.

## Methods

A postal questionnaire survey of all general practices in a single health region in the West of Ireland (County Mayo; total population 123,839 in 2006 Census) was carried out [3]. Ethical approval was granted by the Ethics committee of the Irish College of General Practitioners (Protocol number: REC08-02). The pre-piloted questionnaire was distributed, along with a cover letter, to all General Medical Service (GMS) registered general practices in County Mayo in July, 2008 (See Additional File 1). Systematic postal and telephone contact was made with non-responders. The data was analysed using SPSS version 15.

## Results

### Study participants

**Figure 1:**
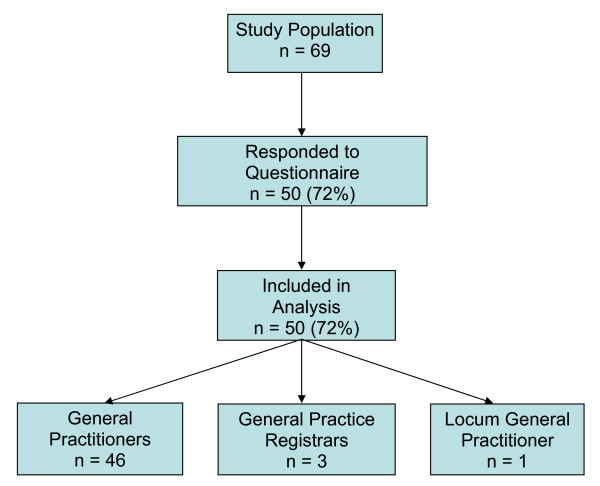
**Participants in the study according to STROBE Statement guidelines**.

All 69 GMS practices in the study area were included in the study and 50 (72%) completed questionnaires were eventually returned and included in the analysis. Figure 1 describes the participants in the study according to the STROBE Statement guidelines [4]. No significant differences were observed among respondents and non-respondents in terms of gender and level of experience. Of the doctors included in the study, 46 were general practitioners, 3 were general practice trainees and 1 was a locum general practitioner. Experience in general practice averaged 21 years, with a range from 1 to 43 years. A large majority (84%) of respondents had less than one year of hospital based paediatric experience and 16% had more than one year. As many as 72% of respondents held the Diploma in Child Health (DCH), but the remaining 28% of doctors had no paediatric qualification. No respondent held the Membership of the Royal College of Physicians in Paediatrics. The majority of respondents (70%) were male and most of respondents (78%) had children of their own.

### Diagnosis

All respondents regarded it important to consider the diagnosis of UTI in all children with unexplained fever. Doctors were asked to identify risk factors for UTI in children from a supplied list of potential indicators. Predisposing factors which were correctly identified by respondents included constipation (72%), family history of vesicoureteric reflux or renal disease (92%), history suggesting previous UTI (100%) and a history of recurrent unexplained fever (92%).

When asked how they would advise parents to collect a urine sample from a one-year-old child, 80% of respondents said that they would advise the use of a urine collection bag while the remaining 20% said they would advise parents to collect a clean catch sample. No respondent said that they would use a urine collection pad. A large majority (81%) of respondents use a urinary dipstick to diagnose urinary tract infection in two-year-old children but 19% do not. Table 1 describes the most common symptoms and signs suggesting urinary tract infection in two-year-old children as identified by respondents.

**Table 1:** Symptoms and signs suggesting UTI in two-year-old children.

	n	Common n (%)	Uncommon n (%)
Fever	49	45 (91.8)	4 (8.2)

Haematuria	47	12 (24.5)	35 (74.5)

Frequency	48	29 (60.4)	19 (39.6)

Dysuria	48	32 (66.7)	16 (33.3)

Abdominal Pain	46	42 (91.3)	4 (8.7)

Offensive Urine	43	22 (51.2)	21 (48.8)

Cloudy Urine	45	22 (48.9)	23(51.1)

Vomiting	48	44 (91.7)	4 (8.3)

Absolute numbers with percentages in brackets.

### Investigation

Respondents were presented with the scenario of a two-year-old child, with a first diagnosis of UTI, who responds well to treatment within 48 hours. Based on this scenario a high degree of variance in their investigative practices was identified (Table 2). Further investigation was seen as necessary by 50% of respondents while the remaining 50% considered it unnecessary. Some 41% stated that such a child would require specialist referral, while 59% did not feel referral was required. Participants were asked if the gender of the child would influence their decision to investigate and/or refer. Some 63% said that it would and the majority of these (84%) were more inclined to refer a boy.

**Table 2:** Investigation and referral patterns. *

	n	Yes n (%)	No n (%)
Further investigation required?	48	24 (50)	24 (50)

Specialist referral required?	46	19 (41.3)	27 (58.7)

Does sex of the child influence decision?	49	31 (63.3)	18 (36.7)

Absolute numbers with percentages in brackets.

* Note: These questions relate to the scenario presented in the 'Investigation' section of the survey questionnaire.

### Management

Respondents were asked to select from a list of antibiotics the ones they would prescribe when treating a child for suspected UTI before culture and sensitivity results were available. They were asked to indicate if they would use each antibiotic 'frequently', 'sometimes' or 'never' for this type of "blind" treatment. Their preferences are presented in Table 3. Some 72% of respondents frequently use co-amoxiclav in this situation. Other antibiotics which respondents report using frequently are amoxicillin (56%) and trimethoprim (49%). Antibiotics which respondents most commonly indicated that they "never used" included ciprofloxacin (73%), erythromycin (57%) and cephradine (57%). Respondents were also asked to indicate how many days of antibiotics they would prescribe in the case of a 6-year old child with a lower urinary tract infection (i.e. cystitis). The answers ranged from 3 to 10 days, with a median duration of 5 days treatment and a mean duration of 5.65 days.

**Table 3:** Doctors' choices of antibiotics for the "blind" treatment of UTI in children.

	n	Frequently n (%)	Sometimes N (%)	Never n (%)
Amoxycillin	43	24 (56)	11 (26)	8 (18)

Ceflaclor	38	8 (21)	16 (42)	14 (37)

Cephradine	37	3 (8)	13 (35)	21 (57)

Ciprofloxacin	37	2 (5)	8 (22)	27 (73)

Co-amoxiclav	44	32 (73)	9 (20)	3 (7)

Erythromycin	37	3 (8)	13 (35)	21 (57)

Trimethoprim	41	20 (49)	12 (29)	9 (22)

Absolute numbers with percentages in brackets.

### Use of guidelines

Some 26% of respondents access clinical guidelines for the diagnosis and management of UTI in children but 74% do not. Of those who do access clinical guidelines, 80% felt the guidelines had influenced their management of UTI in children.

## Discussion

### Summary of main findings

The most striking finding of this study is the clear variation in practice between general practitioners with regard to the investigation and specialist referral of children with UTI. Furthermore, this study demonstrates that there is a clear practice of prescribing courses of antibiotics which are appreciably longer than the recommended three days of treatment for uncomplicated lower UTI's. The study also demonstrates that general practitioners are familiar with the risk factors for UTI in children and that they are aware of the common symptoms and signs of UTI in children.

### Comparison with the NICE guideline and existing literature

It has been shown that childhood UTI's are often not recognised by general practitioners [5]. Under-diagnosis of UTI in children is thought to be responsible for a significant number of patients developing end-stage renal failure as a consequence of acquired renal scarring [6-8]. In the past, children with a confirmed UTI were thoroughly investigated so that any underlying predisposing cause was established. The new NICE guidelines place much less emphasis on advanced investigation. Their main thrust is to ensure that all children with UTI are correctly diagnosed and appropriately treated. The guidelines suggest this approach may be more effective in preventing acquired renal scarring. Thus one of the main objectives of the new NICE guideline is to encourage general practitioners to consider the diagnosis of UTI at an early stage when assessing a sick child. It is most encouraging to note that all of the doctors in this study agreed that it was important to consider the diagnosis of UTI in all children with unexplained fever.

This survey revealed that doctors accurately identified common symptoms and signs of UTI. Several studies have reported on symptoms and signs in children presenting with UTI to a hospital setting [9-19]. Two studies have looked specifically at children presenting to a general practice [20,21]. In a preverbal child, fever is consistently the most common symptom. Verbal children, like adults, most commonly present with dysuria and frequency. Other common symptoms include abdominal pain, loin tenderness, vomiting and poor feeding. The majority of doctors in this study identified the appropriate common symptoms and signs (Table 1).

The NICE guideline places an emphasis on recording the presence of risk factors for UTI and serious underlying pathology. In this study, when doctors were given a list of potential risk factors, they were able to accurately identify 87% of the relevant risk factors for UTI. This demonstrates a high level of awareness amongst doctors of the relevant risk factors for UTI and underlying pathology. When compared with existing similar literature, doctors in this study demonstrated a superior knowledge of predisposing factors for UTI [22]. One risk factor which was insufficiently recognized was constipation, with 17% of respondents indicating that this was not a risk factor for UTI.

Collection of an appropriate urine sample is an important component of the accurate diagnosis of urinary tract infection in children. It remains a challenging process, especially in children who are not toilet trained. Jadresic *et al *showed that the more general practitioners send urine samples from children, the higher the diagnostic rate in that practice [23]. The NICE guideline suggests that general practitioners advise parents to collect a clean catch sample where possible. This recommendation is mainly based on a systematic review which identified five studies that compared the diagnostic accuracy of clean catch urine samples with that of urine samples obtained by supra-pubic aspirate (SPA) [24]. In general, the diagnostic accuracy of the clean catch samples was comparable to that of the SPA samples. In this study, most doctors said that they would advise parents to use a urine collection bag. It is possible that this preference is in part driven by parental choice. In one study that examined parental preferences for collecting a urine sample at home from an infant, the majority of parents found collection of a clean catch urine to be time-consuming and often messy [25]. No respondent in this study indicated that they would use a urine collection pad to collect a urine sample from an infant. The NICE guideline reports insufficient evidence to recommend a preference for the use of pads or bags. It is noted, however, that pads are considerably less expensive, and, based on cost considerations, their use is recommended in the guideline. It has been shown also that parents find pads easier to use and more convenient than urine collection bags [25].

The NICE guidelines recommend the use of dipstick testing only in the case of children over the age of three years. The diagnostic accuracy of leukocyte esterase and nitrite dipsticks is much lower in younger children [26]. There is a considerable risk of missing a proportion of cases of acute UTI in infants and children younger than three years when using dipstick testing, as frequent bladder emptying leads to a lack of urinary nitrite [27]. It is most interesting that 81% of doctors in this study indicated that they would use a urinary dipstick to help diagnose a UTI in two-year-old children. This may represent a situation where it is not feasible to give practical effect to evidence based guidelines, because there is no other test for UTI that provides immediate results unless doctors can carry out or access microscopy. This is acknowledged in the guidelines, with a suggestion that a urinary dipstick could be used for a relatively well child under the age of three years, with non-specific symptoms, provided the test is backed up by non-urgent microscopy.

This study demonstrates a clear variation in practice, amongst doctors working in primary care, with regard to investigation and specialist referral of children with UTI. In general the NICE guidelines recommend against detailed investigation and specialist referral of children with their first diagnosis of UTI, who respond well to treatment within 48 hours and have no atypical features. According to NICE, gender is no longer a major factor in influencing the decision to refer or investigate children with UTI. However, this study reveals that practice is gender dependent, with most respondents having a lower threshold for investigating and referring a boy.

In this study, co-amoxiclav was the antibiotic most commonly prescribed, by general practitioners for the 'blind' treatment of UTI in children. This is consistent with the results of a large Dutch family practice cohort study [28]. Three randomized control trials which compared the effectiveness of different oral antibiotics in lower UTI in children reported no significant difference between treatments [29-31]. NICE do not recommend a specific antibiotic for 'blind' treatment, but instead suggest that the choice should be based upon locally developed multidisciplinary guidance. It is suggested that an antibiotic with low resistance patterns, such as a cephalosporin or co-amoxiclav, should be used when treating an upper urinary tract infection with oral antibiotics. It is also suggested that trimethoprim, nitrofurantoin, cephalosporin or amoxicillin may be suitable for the treatment of lower urinary tract infection (cystitis). At a local level, the microbiology department servicing the study region recommends co-amoxiclav as a first line treatment for uncomplicated lower urinary tract infection. This recommendation arises from the increasing resistance to *E. Coli *in this region, as well as increasing trimethoprim resistance rates which are currently over 20% in this laboratory catchment area.

There was a wide variation in the number of days of antibiotic treatment which doctors prescribe for a lower UTI in a six-year-old child. A Cochrane review which included 10 randomized control trials comparing short (2-4 days) with standard (7-14 days) duration of oral antibiotic is quoted in the NICE guideline. There was no significant difference between the two groups to justify the longer duration of therapy [32]. NICE clearly recommends treatment with oral antibiotics for three days for children aged over three months with lower UTI. It also recommends that parents and carers should be advised to bring the child back to the general practitioner for reassessment if the child is still unwell after 24-48 hours.

The wide variation in the practice of general practitioners reported in this study is in keeping with the findings of similar studies in other countries [28,33]. However, it is important to note that this study was carried out 10 months after the publication of a significant clinical guideline on the subject. Thus, it was anticipated that this study might have demonstrated a more consistent approach by general practitioners to UTI in children. However, only minority of respondents indicated that they had accessed clinical guidelines on the subject.

### Strengths and limitations of this study

As far as can be ascertained, this is the first investigation of general practitioners' management of UTI in children in Ireland. It is important to note that the present study specifically investigated how general practitioners' follow the NICE guideline in their approach to UTI in children. Although all general practices in the region were included in the study, the study sample was small. However, extensive follow-up effort yielded a promising response rate of 72%.

### Implications for future research and clinical practice

This research reveals that doctors working in primary care appropriately consider the diagnosis of UTI in children with unexplained fever. They also demonstrate a high level of awareness of relevant risk factors for UTI in children. There is a clear preference for the collection of urine samples using urine collection bags, whereas evidence based guidelines recommend the use of a clean catch technique. There is considerable variation in practice amongst doctors with regard to the detailed investigation and specialist referral of children with UTI. Clarification is needed as to whether they should follow the NICE guidelines more closely in this regard. Consideration should be given to further research to establish the reasons for such a variation in practice. Co-amoxiclav is the most frequently prescribed antibiotic for UTI and this is an appropriate choice when local resistance patterns are considered. However, there is a clear practice of prescribing courses of antibiotics which are appreciably longer than the recommended three days of treatment for uncomplicated lower UTI's.

## Conclusions

This research highlights the considerable variation in the management of UTI in children in primary care in the context of the NICE guideline on the subject. We suggest that a more standardized approach, as recommended in the NICE guideline, could help general practitioners to improve speed and accuracy of diagnosis and treatment of UTI and thus prevent serious sequelae.

## Competing interests

The authors declare that they have no competing interests.

## Authors' contributions

KK collected and analysed the data and led the write up. LG and BD contributed to the design of the study, data analysis and write up. All authors have read and approved the final manuscript.

## Pre-publication history

The pre-publication history for this paper can be accessed here:

http://www.biomedcentral.com/1471-2296/11/6/prepub

## Supplementary Material

Additional file 1 **Questionnaire for postal survey**. Questionnaire used for the postal survey of the management of urinary tract infection in children in primary care. Click here for file
