# Supplementary material for: A survey of the management of urinary tract infection in children in primary care and comparison with the NICE guidelines
Source: BMC Fam Pract. 2010 Jan 26;11:6. doi: 10.1186/1471-2296-11-6 (PMC2823660; doi:10.1186/1471-2296-11-6)
Supplement: Additional file 1 — Questionnaire for postal survey. Questionnaire used for the postal survey of the management of urinary tract infection in children in primary care. [file 1471-2296-11-6-S1.DOC]

Evaluation of General Practitioners approach to Urinary Tract Infection in Children less than 16 years of age

Demographics

• Are you a GP 🞏 GP Trainee 🞏 Other 🞏

Specify_______________

• For how many years have you worked in general practice? ________

• Do you have hospital based paediatric experience of 1 year or less 🞏

More than1 year 🞏

• Please indicate if you hold either of these qualifications: DCH 🞏

MRCPI (paediatrics) 🞏

• Are you Male 🞏 Female 🞏

• Do you have children? Yes 🞏 No 🞏

Diagnosis

• Please indicate your level of agreement with the following statement:

“It is important to consider the diagnosis of UTI in all children with unexplained fever”

Agree 🞏 Neutral 🞏 Disagree 🞏

• Predisposing factors for UTI in children include:

Constipation Yes 🞏 No 🞏 Not sure 🞏

Poor growth Yes 🞏 No 🞏 Not sure 🞏

Family history of vesicoureteric reflux or renal disease Yes 🞏 No 🞏 Not sure 🞏

History suggesting previous UTI Yes 🞏 No 🞏 Not sure 🞏

Recurrent unexplained fever Yes 🞏 No 🞏 Not sure 🞏

• When asking parents to collect a urine sample from their **1-year old child**, which method would you advise them to use? (please tick one box only)

Pad 🞏

Bag 🞏

Clean catch 🞏

Other (please specify) ______________

• Would you use a urinary dipstick to diagnose urinary tract infection in **2-year old children**? Yes 🞏 No 🞏

Any comment? ____________________________________________________________

• Which symptoms and signs suggest urinary tract infection to you in **2-year old children?** (please tick a box for each symptom)

Common Uncommon

Fever 🞏 🞏

Haematuria 🞏 🞏

Frequency 🞏 🞏

Dysuria 🞏 🞏

Abdominal pain 🞏 🞏

Offensive urine 🞏 🞏

Cloudy urine 🞏 🞏

Vomiting 🞏 🞏

Other_________________

Investigation

• A **2-year old child,** with a first diagnosis of UTI, responds well to treatment within 48 hours:

Does this child require further investigation? Yes 🞏 No 🞏

Dose this child require specialist referral? Yes 🞏 No 🞏

Does the sex of the child influence your decision to investigate/refer? Yes 🞏 No 🞏

If yes, would you be more inclined to investigate/refer a boy or a girl? Boy 🞏Girl 🞏

Management

• When treating a child for UTI before culture and sensitivity results are available (i.e. ‘blind’ treatment), which antibiotic(s) would you commonly prescribe?

Amoxycillin (Amoxyl) Frequently 🞏 Sometimes 🞏 Never 🞏

Ceflaclor (Distaclor) Frequently 🞏 Sometimes 🞏 Never 🞏

Cephradine (Velosef) Frequently 🞏 Sometimes 🞏 Never 🞏

Ciprofloxacin (Ciproxin) Frequently 🞏 Sometimes 🞏 Never 🞏

Co-amoxyclav (Augmentin) Frequently 🞏 Sometimes 🞏 Never 🞏

Erythromycin (Erythroped) Frequently 🞏 Sometimes 🞏 Never 🞏

Trimethoprim (Monotrim) Frequently 🞏 Sometimes 🞏 Never 🞏

Other___________________ (name)

• When treating a **6-year old child** for lower urinary tract infection (cystitis), how many days of antibiotics do you prescribe?

______ days (please specify) Any comment? _______________

• In a **6-year old child** with clinical evidence of a UTI and urinary dipstick is leukocyte positive and nitrite negative, do you (please tick one box only)

Send urine for microscopy and culture 🞏

Start antibiotic treatment 🞏

Both of the above 🞏

Guidelines

• Do you access clinical guidelines for the diagnosis and management of UTI in children? Yes 🞏 No 🞏

• If you access clinical guidelines, please provide details (name, source, etc...) ­

­­­­­­­­­­ _________________________________________________________________

• Have these guidelines changed your management of UTI in children and if so, how?

Yes 🞏 No 🞏

How?_____________________________________________________________________________________________________________________________________

• Any further comments on diagnosis, treatment and long-term management of UTI in children?

_________________________________________________________________

­­­­­­_________________________________________________________________

Return to Dr Kieran Kennedy, The Medical Centre, Knock, Co. Mayo.
